# Determination of the Physical and Chemical Properties and Volatile Aroma Components Distribution of Geographically Indicated Besni Grape Raisin

**DOI:** 10.1002/fsn3.72183

**Published:** 2026-08-08

**Authors:** Fatma Erişik, Nizam Mustafa Nizamlioğlu, Yalçın Güçer

**Affiliations:** ^1^ Department of Food Engineering, Faculty of Engineering Karamanoglu Mehmetbey University Karaman Turkey; ^2^ Department of Culinary, Beypazarı Vocational School Ankara University Ankara Türkiye

**Keywords:** aroma component, ATR‐FTIR, Besni, quality characteristics, raisin

## Abstract

In this study, the pH, titration acidity, °Brix, color, and moisture values, as well as the volatile aroma profiles of yellow and black grapes obtained from the Besni and Gölbaşı districts of Adıyaman—dried either on the branch or in the sun—were evaluated based on region and drying method. The pH, titration acidity, °Brix, and moisture values of the raisin varieties ranged from 4.017 to 4.440, 1.263 to 1.760 g/L, 63.333% to 71.667%, and 7.610% to 10.160%, respectively. Hunter L*, a*, and b* color values were determined in the ranges of 14.117 to 23.677, 1.055 to 6.853, and 0.583 to 10.860, respectively. A total of 73 volatile aroma compounds were identified in the raisins using the HS‐SPME‐GC–MS method, with their distributions calculated as relative peak‐area percentages. Among these, 5‐(hydroxymethyl)furan‐2‐carbaldehyde (HMF), ethyl ethanoate, and propane‐1,2,3‐triyl triacetate were detected in all raisin varieties. The color and aroma profiles of Besni grapes were found to exert a decisive effect on overall quality. Direct sun drying significantly affected the pH and moisture content, particularly in black raisins. According to the Nested Analysis of Variance (Nested ANOVA), geographical origin had no significant influence on the physical and chemical properties of the raisins, whereas the choice of post‐harvest drying treatment had an incredibly significant impact on final product quality.

## Graphical abstract

This study characterized the physical, chemical, and volatile aroma profiles of geographically indicated yellow and black Besni grapes harvested from the Besni and Gölbaşı districts of Adıyaman, Türkiye. The effects of on‐branch dehydration versus traditional direct sun drying were comprehensively evaluated. While geographical origin showed no significant variance (*p* > 0.05)—confirming a uniform regional terroir—the post‐harvest drying method played a transformative role (*p* < 0.01) in shaping moisture content, pH, color, and aroma complexity (73 identified volatile compounds). Furthermore, non‐destructive ATR‐FTIR spectroscopy integrated with Partial Least Squares Regression (PLS‐R) proved to be an accurate, chemical‐free tool for predicting moisture and titratable acidity in raisin matrices.

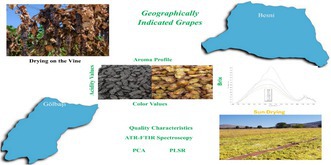

## 1 Introduction

Türkiye is considered a major player in the global viticultural landscape, owing to the favorable ecological and climatic conditions present in the country. The development of appropriate cultivation techniques has been enabled by the existence of climate differences, and consumption and utilization methods have diversified (Balbaba and Bağcı 2021). With a vineyard area of 600.000 ha, Turkey ranks 5th globally in terms of vineyard area and 6th in terms of fresh grape production (Akgün and Akgün 2006; Onar 2013). The consumption patterns of Turkish grapes are diverse, with table grapes and dried grapes being the most widely consumed types. However, the utilization of grapes in the production of various products, including sucuk (a type of sausage), pestil (fruit leather), pekmez (grape molasses), wine, and şıra (unfermented grape juice), is also significant (Onar 2013).

According to data from TÜİK (2021), Turkey produced approximately 4.2 million tons of grapes in 2021, with 52.8% being table grapes, 36.5% dried grapes, and 10.7% wine grapes. The drying process applied to fruits is intended to inhibit the activity of certain microorganisms. This process serves to extend the shelf life of fruits, enabling their consumption over a more protracted period. The best and quickest way to preserve fresh grapes is by removing water, that is, dehydration lowers the water activity and thus naturally prevents the fruit from deterioration. Further, the weight and volume of grapes reduce, which facilitates low transportation cost, convenience in handling, extra income, and ease of bulk storage (Patidar et al. 2021). Dried grapes have valuable nutritional properties because of high mineral, vitamin, flavonoids, anthocyanins, and other bioactive substance content. Therefore, current research indicates that grape consumption has many health benefits such as the reduction of the risk of cancer, type 2 diabetes, stroke, obesity, cardiovascular diseases, and other chronic diseases (Langová et al. 2020; Patidar et al. 2021). The ‘Sultani Seedless Grape’ variety is the most produced seedless dried grape variety in Turkey, with an annual production of 250,000 to 300,000 tons (Uysal Seçkin 2019). The most prominent seeded dried grape varieties include Besni, Rumi, Göğüzüm, Dımışkı, Sergi Karası, Horoz Karası, Ekşi Kara, Banıma Siyahı, Kerküş, Zeynebi, and Kara Dimrit (Mazlum 2020).

Drying processes considerably affect the raisin quality due to their impact on the duration of dried, sugar concentration, and enzymatic activity (Keskin et al. 2022). Different techniques, depending on the geographic location and grape variety, produce dried grapes or raisins. These techniques include open drying, mechanical drying, and shade drying (Natarajan et al. 2024). To shorten the drying time and reduce the contamination risk, different methods such as solar, oven, and hot‐air drying could be used in raisin production. Sensorial properties, nutritional quality, and cost of product are usually taken into consideration in choosing an appropriate drying method (Çoklar and Akbulut 2017). Grapes are susceptible to drying and are traditionally dried in the sun for 8–10 days. This method of drying is cheap, but there is a risk of the grapes being infested by insects or contaminated (Langová et al. 2020). In sun drying, the grapes are traditionally laid on racks or trays on the ground to be exposed to natural air and the sun for 2 or 3 weeks (Keskin et al. 2022). The drying method on the branch involves drying the grapes on the vine before they are harvested.

The choice of post‐harvest dehydration methodology plays a transformative role in shaping the ultimate quality, flavor complexity, and compositional profile of raisins. Traditional standard detached drying involves harvesting clusters at optimum maturity and exposing them directly to solar radiation on ground trays or racks. This physical detachment abruptly halts the influx of vascular sap, subjecting the grape berry to immediate systemic tissue shock. The sudden isolation triggers rapid moisture loss through the cuticle, inducing severe cellular desiccation that accelerates cell wall degradation, vacuolar collapse, and the chaotic mixing of localized enzymes and substrates. In stark contrast, drying grapes directly on the branch—wherein clusters remain physically connected to the living vine—extends a dynamic, controlled physiological state during the initial phases of dehydration. Maintaining vascular continuity via the functional xylem and phloem radically modulates cell respiration, moisture loss kinetics, and metabolic pathways (Bellincontro et al. 2004; Sanmartin et al. 2021).

On‐branch drying subjects the attached fruit to prolonged, deliberate environmental stressors, such as ambient temperature fluctuations and ultraviolet radiation, while keeping systemic signaling pathways intact (Rienth et al. 2021). In response to this gradual, non‐lethal stress, the berry activates secondary metabolic defense mechanisms. This can shift phenylpropanoid pathways, triggering the synthesis and accumulation of protective secondary metabolites—including polyphenols, flavonoids, and specific volatile aroma precursors (such as bound glycosidic esters and terpenes)—before final cellular activity ceases (Degu et al. 2021; Rienth et al. 2021).

The province of Adıyaman, particularly the district of Besni, is known for its extensive grape cultivation, and the dried grape trade holds significant economic importance in this region (Özkaya et al. 2014). The Besni grape, a prominent variety, is extensively traded in the surrounding regions (Özkaya et al. 2014). The drying process is initiated once the grapes have reached their optimal maturity level (Yalçınkaya 2016). Previous studies have shown that agronomic practices (leaf removal, training system, foliar fertilization, water deficit, exogenous spraying, and cluster thinning) can influence the precursors of aroma metabolism in grapes, thereby affecting the release of aromas (Wang et al. 2024).

Attenuated total reflectance (ATR) Fourier‐transform infrared spectroscopy (FTIR) has been widely used in the analysis of fruits and vegetables (Sarkar et al. 2021; Nizamlıoğlu et al. 2022). For a reliable and non‐destructive measurement in fruit and vegetable analysis, FTIR spectroscopy can be combined with quantitative and qualitative chemometric techniques, specifically Pearson's correlation and regression models like principal component analysis (PCA) and partial least squares regression (PLSR) (Bureau et al. 2019). In comparison to several chemical methods, a successful use of the FTIR spectro‐chemometric method for determining the nutritional composition of food has been documented (Nizamlıoğlu et al. 2022).

Although previous studies have thoroughly evaluated the physical and chemical attributes of standard sun‐dried seedless cultivars—most notably the Sultani Seedless grape—and explored the impact of pre‐harvest agronomic practices on grape aroma precursors, a profound literature gap remains regarding the spectrochemometric and volatile characterization of indigenous, geographically indicated seeded varieties undergoing on‐branch dehydration. Crucially, the simultaneous evaluation of regional environmental commonalities and divergent drying practices using advanced multivariate modeling has not been adequately established for traditional Anatolian cultivars. To bridge this knowledge gap, the present study comprehensively investigates the physical, chemical, and volatile aroma profiles of geographically indicated Besni grapes obtained from the Besni and Gölbaşı districts of Adıyaman, systematically evaluating the distribution of free aroma substances under both on‐vine and direct sun‐drying treatments. Furthermore, this research introduces a distinct novelty by establishing non‐destructive ATR‐FTIR spectroscopy coupled with Partial Least Squares Regression (PLS‐R) and Taguchi optimization workflows to precisely quantify and model quality attributes in these specialized matrices. By cross‐validating rapid spectrochemometric fingerprints against gold‐standard chemical and chromatographic data, this study serves as a predictive benchmark to safeguard product typicity, standardize traditional post‐harvest practices, and optimize quality control frameworks across the regional raisin industry.

## 2 Materials and Methods

### 2.1 Material

The Besni dried grape samples utilized in this study were obtained from commercial vineyards situated in the Besni and Gölbaşı districts of Adıyaman province. To ensure authentic regional representation and eliminate sampling ambiguity, three distinct local producers were selected as independent biological replication sources within each district, totaling six unique vineyard sites (*n* = 6). Mature grape clusters harvested from each independent producer were subsequently split and subjected to two divergent post‐harvest dehydration methods (branch drying and sun‐drying). Within these drying treatments, the grape samples were further categorized based on their natural pigmentation profiles, encompassing both Black and Yellow variants. This hierarchical experimental setup generated six distinct sample combinations. This hierarchical experimental setup generated six distinct sample combinations. It must be noted as an inherent experimental constraint that yellow grape variants dried on the branch (BYB and GYB) could not be obtained due to regional agricultural limitations and localized harvest conditions during the production season. Consequently, the branch‐drying treatment group is exclusively represented by the black grape variants (BBB and GBB). This structural imbalance within the experimental design was fully accounted for during downstream multivariate data modeling and partition tracking. All physical and chemical analyses were performed in technical triplicate for each biological sample. For the chemometric calibration and validation workflows, a representative subset of 30 independent ATR‐FTIR spectral scans was compiled from the dried grape matrices. These spectra were systematically partitioned, randomly allocating 24 baseline‐corrected spectra to construct the Partial Least Squares Regression (PLS‐R) calibration models and reserving the remaining 6 spectra as an independent validation set to rigorously assess the predictive accuracy and reliability of the spectrochemometric method. This structured sampling density provides a statistically adequate number of degrees of freedom to validate the downstream Factorial ANOVA, Nested ANOVA, Principal Component Analysis (PCA), and PLS‐R predictive validations reported herein. The samples nomenclature and factorial experimental design matrix is provided in Tables 1 and 2.

**TABLE 1:** Populated cells of the factorial experimental design matrix.

Growing region	Grape color	Post‐harvest drying method	Sample abbreviation	Status/biological replications
Besni District	Black	On‐Branch Dehydration	BBB	Populated (*n* = 3 independent producers)
	Black	Direct Sun Drying	BBS	Populated (*n* = 3 independent producers)
	Yellow	Direct Sun Drying	BYS	Populated (*n* = 3 independent producers)
	Yellow	On‐Branch Dehydration	—	Unpopulated Cell (Agricultural Constraint)
Gölbaşı District	Black	On‐Branch Dehydration	GBB	Populated (*n* = 3 independent producers)
	Black	Direct Sun Drying	GBS	Populated (*n* = 3 independent producers)
	Yellow	Direct Sun Drying	GYS	Populated (*n* = 3 independent producers)
	Yellow	On‐Branch Dehydration	—	Unpopulated Cell (Agricultural Constraint)

*Note:* Each populated cell represents 3 independent biological vineyards (*n* = 6 total vineyard sites across both districts). All physical and chemical laboratory analyses were performed in technical triplicate for each biological sample. As explicitly outlined, the yellow branch‐dried cells remain unpopulated due to regional harvesting constraints and localized agricultural availability during the production season.

**TABLE 2:** Black and yellow grape varieties from Besni and Gölbaşı.

Sample abbreviation	Growing region	Grape color	Drying method
BBB	Besni	Black	Branch
BBS	Besni	Black	Sun
BYS	Besni	Yellow	Sun
GBB	Golbası	Black	Branch
GBS	Golbası	Black	Sun
GYS	Golbası	Yellow	Sun

### 2.2 Moisture Determination

Moisture analysis was performed in triplicate using an electronic moisture analyzer (OHAUS MB45, Switzerland) (Nizamlıoğlu 2015; Jimoh et al. 2024).

### 2.3 Determination of Soluble Solid Content (°Brix)

°Brix in dried grape samples was determined using a benchtop refractometer. Prior to measurement, the refractometer was calibrated with distilled water (AOAC 1984). Raisin samples (5 g) were mixed with 45 mL distilled water in 100 mL containers, stirred intermittently until homogeneous, and allowed to equilibrate for 4 h. The °Brixvalue in the original sample was then calculated by considering the dilution ratio of the dried grape samples (Cemeroğlu 1992).

### 2.4 Determination of Color Values

The color of the dried grape samples was determined using a color measurement device (CHROMA METER CR‐400, Konica Minolta, INC., Japan). Prior to use, the device underwent a calibration process (Birch et al. 2010). The color values measured in the grape samples included L* (lightness), a* (green to red variation), and b* (blue to yellow variation) (Nizamlıoğlu 2015).

### 2.5 pH Determination

The pH value was determined potentiometrically using a digital pH meter with a glass electrode following the method suggested by Cemeroğlu (2010). Prior to measurement, the pH meter was calibrated with buffer solutions at pH 4 (highly acidic) and pH 7 (neutral) (Cemeroğlu 2010).

### 2.6 Determination of Titratable Acidity

The titratable acidity values were determined by titration of the samples with a 0.1 N NaOH solution, and the results were expressed in grams of tartaric acid per liter (Nelson 1985). In the titration acidity method, 20 mL of ultrapure water was added to 10 mL of dried grape filtrate, and the mixture was titrated with 0.1 N NaOH until the pH reached 8.1 (Otağ 2015).

### 2.7 Aroma Components Analysis

The volatile aroma components of the dried grape samples were determined using the HS‐SPME method. Following the extraction of the aroma components from the dried grape samples, they were adsorbed onto a fiber and analyzed using a GC–MS device (Shimadzu, Japan). The relative distribution of the volatile aroma compounds was expressed as relative peak‐area percentages calculated by normalizing individual peak areas against the total chromatographic peak area; no internal standard was utilized for absolute quantification. A 3 g sample was weighed into 20 mL headspace vials, and the vial's mouth was sealed. Then, the fiber (85 μm thick carboxen/polydimethylsiloxane) was immersed in the sample vial, and the volatile components in the headspace of the vial were captured by adsorption, and the components were transferred to the GC–MS system by desorption. The analysis of aroma components was performed using a DB‐WAX capillary column (60 m x 0.25 mm x 0.4 μm; J&W Scientific, Folsom, USA) on the GC–MS device (He et al. 2025). The column temperature was initially set at 40°C for a period of 2 min. Thereafter, the temperature increased at a rate of 3°C/min until reaching 80°C, where it was held for 1 min. Thereafter, the temperature increased at a rate of 5°C per minute until reaching 240°C, at which point it was maintained for 6 min. Finally, the temperature was raised to 250°C. Helium gas was selected as the carrier gas, and the flow rate was set at 1.0 mL/min (Xiao et al. 2025). Qualitative methods included matching the spectra with those found in the NIST Mass Spectral Library and combining them with an artificial map spectral analysis (Koprivnjak et al. 2002; Xue et al. 2023; Gao et al. 2021; Deng et al. 2021).

### 2.8 ATR‐FTIR Analysis

The ATR‐FTIR device was utilized to ascertain the material type of the samples and to observe changes after the process. The dried grape samples were subjected to Fourier Transform Infrared Spectroscopy (FTIR) to measure the spectra. The absorption spectra of the samples were obtained using FTIR spectroscopy with an ATR cell (VERTEX 70 Series, Bruker, Germany) located at the Karamanoglu Mehmetbey University Laboratory. A 0.5 cm^3^ portion of each sample was placed on a multi‐reflection ZnSe crystal, and 128 scans were performed at a resolution of 2 cm^−1^ in the range of 400 to 4000 cm^−1^. The spectral data obtained from the samples were then baseline corrected using “0” as a reference, and normalization transformations were applied based on the highest absorbance value (Yasar et al. 2020). All the data optimizations and transformations were performed by using an academic version of Speaktragryph version 1.2.14, kindly provided by Dr. Friedrich Menges, Germany.

To ensure the strict mathematical validity of the Partial Least Squares Regression (PLS‐R) models and eliminate any risk of data leakage within our limited sample cohort, a group‐blocked validation approach was enforced. Although technical triplicates were performed for each treatment, the partitioning of the 30 independent ATR‐FTIR spectral scans into the calibration set (*n* = 24) and the independent validation set (*n* = 6) was executed strictly at the biological sample level rather than the technical replicate level. Consequently, all technical replicates originating from a specific biological sample were assigned as an indivisible block exclusively to either the calibration matrix or the validation set. This structural boundary guarantees that the model was validated against entirely unseen biological profiles, preventing the artificial inflation of the correlation coefficients (Rc and Rcv) and providing an uncompromised evaluation of the spectrochemometric methodology.

### 2.9 Statistical Analysis

The data obtained were evaluated using the analysis of variance (ANOVA) technique with a factorial experimental design (Minitab 1991). The results of the chemical analyses were also assessed using the same technique, and differences between groups were determined by Tukey's Multiple Comparison Test. Three distinct analysis methods were employed to evaluate the results: Principal Component Analysis (PCA), Nested Design Analysis of Variance, and the Taguchi Optimization Method.

## 3 Result and Discussion

### 3.1 Results of Acidity Analysis

It was determined that the pH of Gölbaşı grapes was higher than that of Besni grapes (*p* < 0.05). As demonstrated in Table 3, the pH values of sun‐dried black grapes from both the Besni and Gölbaşı districts were found to be elevated, with values of 4.33 and 4.44, respectively. The findings indicate that direct sun drying exerts a significant effect on the pH of black grapes. These findings indicate that direct sun drying exerts a significant effect on the pH of black grapes, which exhibit a tight metabolic correlation with the observed titratable acidity values.

**TABLE 3:** Acidity, Brix, and moisture results of black and yellow grape varieties from Besni and Gölbaşı districts dried on the vine and in the sun.

Grape variety	pH	Titratable acidity (T.A.) (%)	Brix (°Bx)	Moisture (%)
BBB	4.203 ± 0.093^bc^	1.760 ± 0.017^a^	63.333 ± 0.577^c^	7.610 ± 0.139^d^
BBS	4.327 ± 0.035^ab^	1.263 ± 0.112^c^	67.000 ± 2.646^bc^	9.140 ± 0.618^b^
BYS	4.017 ± 0.0169^d^	1.287 ± 0.162^c^	69.667 ± 1.528^ab^	9.017 ± 0.245^b^
GBB	4.127 ± 0.090^cd^	1.397 ± 0.042^bc^	70.333 ± 1.526^ab^	8.040 ± 0.211^cd^
GBS	4.440 ± 0.040^a^	1.613 ± 0.047^ab^	66.333 ± 0.578^bc^	10.160 ± 0.082^a^
GYS	4.317 ± 0.051^ab^	1.310 ± 0.076^c^	71.667 ± 1.527^a^	8.163 ± 0.130^bc^

*Note:* The means marked with different letters in the same column are statistically (*p* < 0.05) different from each other.

Abbreviations: BBB, Besni Black Branch; BBS, Besni Black Sun; BYS, Besni Yellow Sun; GBB, Golbası Black Branch; GBS, Golbası Black Sun; GYS, Golbası Yellow Sun.

The titratable acidity values of Gölbaşı and Besni grapes were found to be very close to each other, and the results were statistically insignificant (*p* > 0.05). However, a statistically significant difference was observed in the titratable acidity values of the two drying methods (*p* < 0.05). As with the pH values, the titratable acidity values were found to be higher in black grapes for both grape varieties. The Besni grape values were found to be the highest (1.76) in black grapes dried on the vine, while the highest value in the Gölbaşı grapes was found in sun‐dried black grapes (1.61).

The biochemical variance in acidity between on‐branch drying and standard detached sun‐drying is governed by the distinct metabolic states of the fruit tissue during dehydration. When grapes are dried directly on the branch, the clusters maintain vascular continuity with the parent vine, allowing cellular respiration to decline progressively rather than abruptly. The grape berry remains a living, metabolically active organ for a prolonged period. To sustain baseline cellular maintenance under slow dehydration stress, the fruit tissue relies on the tricarboxylic acid cycle as its primary energy engine. This ongoing respiratory consumption of organic acids under on‐branch conditions explains why Besni black grapes dried on the vine exhibited a distinct acidity profile, balancing internal osmotic potential through gradual, regulated vacuolar depletion.

The elevated pH (and altered organic acid concentrations) observed in the direct sun‐dried black grapes is heavily driven by the kinetics of high‐intensity solar radiation and thermal stress. Direct exposure to the sun accelerates moisture loss, causing a rapid concentration of solutes within the cellular matrix. However, the accompanying thermal absorption—which is significantly more pronounced in black grapes due to the high light‐absorption capacity of anthocyanin pigments in the skin—elevates the internal berry temperature. The combination of rapid desiccation and direct UV radiation damages the mitochondrial membrane system, abruptly shifting cellular respiration from aerobic pathways to anaerobic fermentation. This membrane degradation leads to vacuolar leakage, mixing organic acids with cellular cations (such as potassium, K^+^). The leaching of potassium ions induces the partial salification of tartaric acid into potassium bitartrate. Because potassium bitartrate precipitates or has a lower dissociation constant than free tartaric acid, the concentration of free hydrogen ions (H^+^) in the intracellular juice decreases directly resulting in the significantly higher pH levels witnessed in the sun‐dried black samples (4.33 and 4.44).

In line with the findings of Balbaba and Bağcı (2021), who reported a pH range of 3.81–4.06 for Besni grapes, Uysal Seçkin (2019) documented pH values for dried Besni grapes ranging from 3.99 to 4.19, contingent on the employed drying method. In a related study, Baiano et al. (2012) reported titratable acidity values ranging from 1.94 g/L to 4.26 g/L in seven different dried grape varieties. Uysal Seçkin (2019) found the titratable acidity in Besni grapes to vary between 2.2 g/100 g and 2.0 g/100 g. Yalçınkaya (2016) reported titratable acidity values in dried Besni grapes ranging between 0.77 g/100 g and 1.05 g/100 g.

### 3.2 Brix Analysis Results

The findings of the study revealed that the °Brixvalue of Gölbaşı grapes was higher than that of Besni region grapes (*p* < 0.05). No statistically significant difference was observed in the °Brix values of black grape varieties dried on the vine and in the sun (*p* > 0.05), while the °Brixvalues of yellow grape varieties were found to be higher. In Besni grapes, the °Brixvalues of sun‐dried grapes were found to be higher, and in Gölbaşı grapes, the °Brixvalues of sun‐dried yellow grapes were higher than those dried using the other two methods. The highest °Brixvalue (71.67) was recorded in sun‐dried Gölbaşı grape varieties (*p* < 0.05). In a separate study, Şen (2014) dried Sultani Seedless grapes from five different districts of Denizli province in a 2.5% potassium carbonate solution and found °Brixvalues ranging between 75.23 °B and 65.43 °B. In a separate study, Konuk (2014) dried grapes from two different regions using three different drying techniques and found °Brixvalues between 46.6 °B and 75.23 °B.

### 3.3 Moisture Analysis Results

The moisture values of the grape varieties from Gölbaşı were found to be higher than those from Besni (*p* < 0.05). In both the Besni and Gölbaşı districts, the highest moisture content was found in sun‐dried black grape varieties. The highest recorded moisture value (10.16%) was observed in sun‐dried Gölbaşı black grape varieties (*p* < 0.05). The findings of this study indicated that direct sun drying had a significant effect on the moisture content, especially in black grapes.

During on‐branch drying, water loss occurs at a highly controlled, uniform rate under ambient shade and natural canopy airflow. This gentle moisture gradient allows the cellular structure of the skin and pulp to collapse concurrently and systematically. As the cells shrink uniformly, the skin retains a semi‐flexible, porous conformation that prevents case hardening. Sun‐drying forces an immediate, hyper‐accelerated rate of surface evaporation due to direct, unshielded solar radiation. In black grape varieties, the high concentration of skin anthocyanins maximizes thermal energy absorption from the solar spectrum, drastically increasing the evaporation rate of free water from the outermost peripheral cell layers. This intense surface desiccation triggers a physical phenomenon known as case hardening.

The outermost skin layers dry so rapidly that the localized proteins and structural polysaccharides cross‐link and denature prematurely, forming a rigid, impermeable hydrophobic crust on the exterior of the raisin. While the surface appears completely dry and structurally stabilized, a substantial fraction of internal bound water becomes locked within the deep medullary pulp tissue. This explains the paradox of why the sun‐dried black grape varieties retained significantly higher residual moisture values (10.16%), as the structural crusting of the skin artificially halted the advanced phase of mass transfer during open solar exposure.

Uzun and Hallaç (2021) observed that the moisture content in Beşiri dried grapes ranged between 8.70% and 16.70%. Guine et al. (2015) found the moisture content to be 14.52% in grapes dried at 50°C using convective drying and 19.43% in sun‐dried grapes. In addition, Mazlum (2020) reported moisture values ranging between 16.33% and 17.16% in grapes dried in the sun, in the shade, and with a potassium carbonate solution.

### 3.4 Color Analysis Results

**FIGURE 1:**
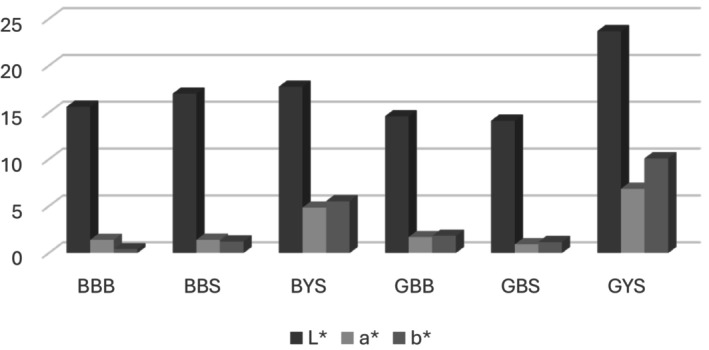
L*, a*, and b* color values of grape varieties. BB, Besni Black Branch; BBS, Besni Black Sun; BYS, Besni Yellow Sun; GBB, Golbası Black Branch; GBS, Golbası Black Sun; GYS, Golbası Yellow Sun.

The L*, a*, and b* color values of the grapes from Gölbaşı were found to be higher than those of the Besni region (*p* < 0.05). In both the Besni and Gölbaşı grape varieties, sun‐dried yellow grape varieties exhibited the highest L*, a*, and b* color values (*p* < 0.05). As demonstrated in Table 4, the highest L*, a*, and b* color values were recorded in sun‐dried Gölbaşı yellow grape varieties, with values of 23.68, 6.85, and 10.86, respectively. These results were statistically significant compared to other grape varieties (*p* < 0.05). As demonstrated in Figure 1, sun‐dried yellow grapes exhibited higher L*, a*, and b* color values compared to black grapes. In a related study, Balbaba and Bağcı (2021) documented L*, a*, and b* color values for fresh Besni grapes ranging between 32.92 and 44.44, (−6.31 and −1.00), and 20.87 and 30.50, respectively. Arpa Zemzemoğlu (2019) determined the L*, a*, and b* color values of Besni grape varieties to be between 23.89 and 36.93, 0.77 and 2.92, and −0.41 and 1.00, respectively. Han et al. (2024) stated that the application of benzothiadiazole on Chardonnay grapes, which are widely cultivated in northwestern China, had a positive effect on sugar content while reducing the amount of chlorophyll and total carotenoids.

**TABLE 4:** Color results of black and yellow grape varieties from Besni and Gölbaşı districts.

Grape variety	L*	a*	b*
BBB	15.597 ± 0.604^cd^	1.147 ± 0.090^c^	0.583 ± 0.045^d^
BBS	16.997 ± 0.555^bc^	1.160 ± 0.130^c^	1.293 ± 0.154^cd^
BYS	17.747 ± 0.658^b^	5.327 ± 0.580^b^	5.857 ± 0.268^b^
GBB	14.587 ± 0.045^d^	0.963 ± 0.74^c^	1.570 ± 0.140^cd^
GBS	14.117 ± 0.566^d^	1.403 ± 0.152^c^	1.960 ± 0.111^c^
GYS	23.677 ± 0.784^a^	6.853 ± 0.664^a^	10.860 ± 0.941^a^

Abbreviations: BBB, Besni Black Branch; BBS, Besni Black Sun; BYS, Besni Yellow Sun; GBB, Golbası Black Branch; GBS, Golbası Black Sun; GYS, Golbası Yellow Sun.

### 3.5 Principal Component Analysis (PCA), Nested Model Variance Analysis, and Taguchi Optimization Method for Physical and Chemical Components of Grape Samples

Principal component analysis (PCA) is a significant method for accurately interpreting the number of principal components that constitute a mixture without requiring detailed information about all the components present. This method plays a crucial role in minimizing errors (Özbay 2022).

**FIGURE 2:**
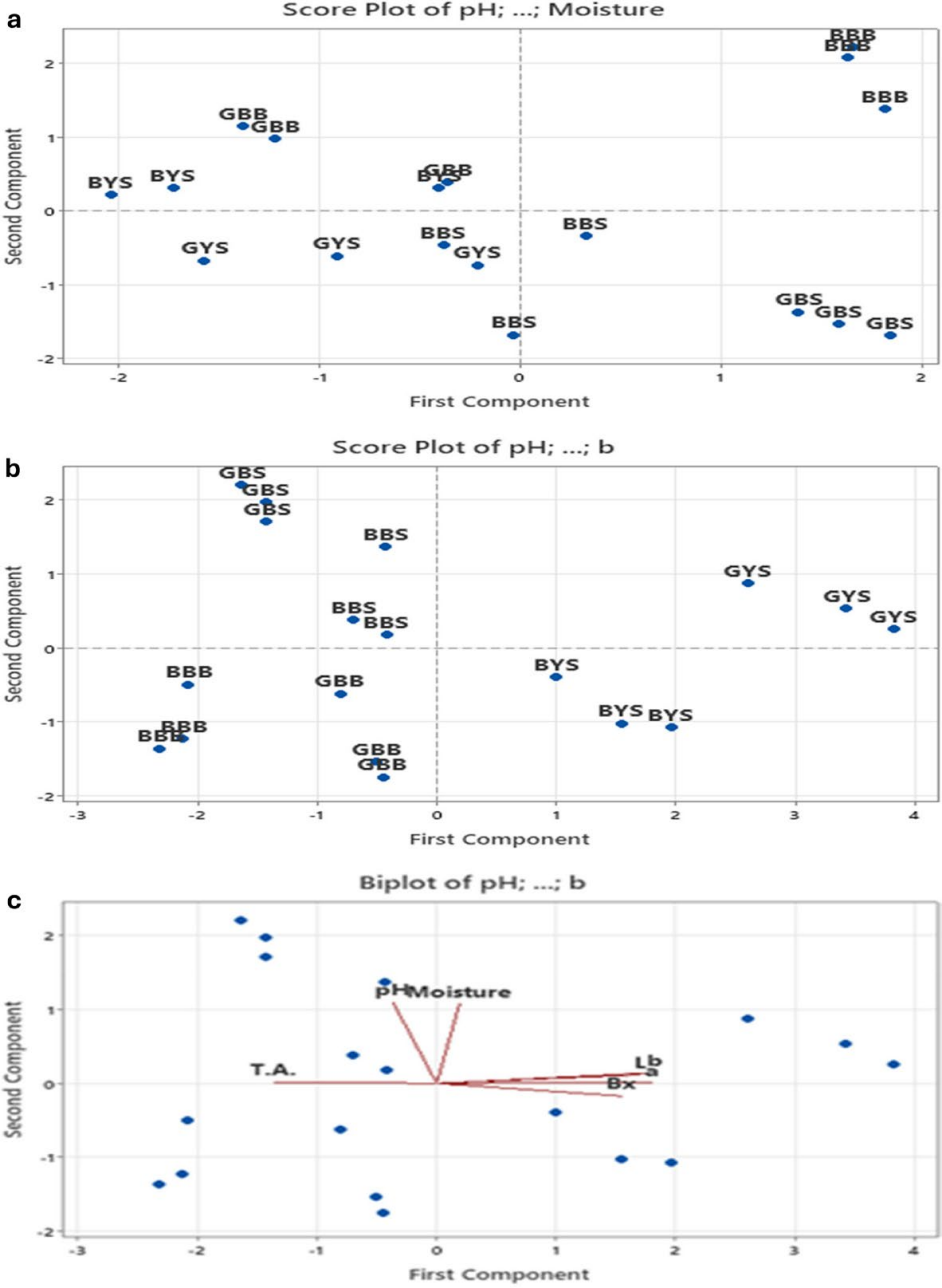
Principal component analysis: (a) Scorplot graph containing pH, Brix, titratable acidity, and moisture (no color values); (b) Scatterplot graph containing pH, Brix, titratable acidity, moisture, L*, a*, and b*; (c) Biplot graph of graph b. BB, Black Branch; BS, Black Sun; YS, Yellow Sun.

As demonstrated in Figure 2, two distinct PCA graphs are presented. The graph on the ‘b’ shows the PCA results based on all physical and chemical analysis results applied to the grape samples, while the graph on the ‘a’ presents the PCA results excluding the Hunter L*, a*, and b* color values. As demonstrated in the PCA graph on the right in Figure 2, where color values are excluded, the BBB‐dried and GBS‐dried grape samples are distinctly positioned away from the other grape samples. Furthermore, these two samples are significantly distant from each other.

In the PCA graph on the ‘b’, which incorporates the Hunter L*, a*, and b* color values, it is evident that color values have a significant effect at the conclusion of all drying processes. In Figure 2 Graph on the left, seven parameters were reduced to 3 components by a PCA. Three components explained an 88.9% of total variance. Distinct distributions are exhibited by different drying methods. As observed Biplot analysis in the lower portion of the graph, the color values and °Brixvalues are situated in the same region as the yellow grape varieties for both types of grapes. This observation is consistent across the drying applications in both regions. Despite regional differences, similar drying methods resulted in closely positioned samples. Consequently, it was determined that the Hunter L*, a*, and b* color values have a significant impact on the quality of dried grape samples. L*, a*, and b* color and °Brixwere the most influencing indicator for sun dried BYS and GYS products.

Nested Analysis of Variance (Nested ANOVA) is a statistical technique that enables the examination of effects and factors within hierarchically structured units at each level. This method facilitates a comprehensive interpretation of production quality by considering each parameter in conjunction with all its combinations (Buyrul 2022). As demonstrated in Table 5, the geographical origin exhibited no substantial influence on the physical and chemical properties (quality) of the grape samples, with the exception of the °Brixvalue. The impact on the °Brixvalue was found to be relatively low at 7.78%. The results of the variance analysis demonstrated that the impact of geographical origin on all components, including the °Brixvalue, was determined to be statistically insignificant. Conversely, disparate drying methodologies were found to exert a substantial influence on product quality across all grape components. The variance analysis results indicated that the effect of the drying method on all components was highly significant (*p* < 0.01).

**TABLE 5:** Variance components of the Nested model in grape samples.

Variance source	pH	°Bx	Titratable acidity	Moisture	L*	a*	b*
Variance components percentage (%)							
Region	0.00	7.78	0.00	0.00	0.00	0.00	0.00
Drying	85.42	70.83	85.72	91.18	97.78	98.35	99.06
Variance analysis *p* (significance levels)							
Region	0.431	0.318	0.987	0.641	0.841	0.833	0.556
Drying	0.000	0.001	0.000	0.000	0.000	0.000	0.000

The variance analysis demonstrates that geographical origin exhibits no substantial influence on the physical and chemical parameters, with the singular exception of the °Brix. Even for °Brix, the regional variance component accounts for a minor 7.78% of total variation. Across parameters like pH, titratable acidity, moisture, and Hunter L*, a*, b* color values, the effect of the geographic origin remains completely insignificant (*p* > 0.05). This mathematically confirms that the baseline physicochemical composition of the grapes harvested from the Besni and Gölbaşı districts is essentially identical. Besni and Gölbaşı are neighboring districts within the Adıyaman province of Turkey. Because they share near‐identical macro‐climatic profiles, elevation ranges, sun exposure hours, and soil compositions, the physiological development of the grapes follows identical metabolic pathways. The Besni grape is a specific genetic variety grown across these adjacent borders. The lack of phenotypic variation in its sugar‐acid ratio, structural moisture capacity, and natural pigments underscores that the raw material behaves uniformly under identical regional conditions.

While the Nested ANOVA cleanly isolates the drying method as the dominant driver of quality characteristics (*p* < 0.01), the lack of statistically significant differences (*p* > 0.05) between the Besni and Gölbaşı districts presents an inherent chemometric paradox. This outcome demonstrates that standard physicochemical parameters (such as pH, titratable acidity, moisture, and color values) cannot serve as definitive, standalone discriminatory markers to separate these two neighboring production areas. However, rather than weakening the Geographical Indication, this regional uniformity mathematically confirms that both districts share an identical agro‐ecological terroir and baseline raw material profile. This establishes that the unique identity of the geographically indicated Besni Grape Raisin is predominantly defined by shared traditional human interventions and processing methodologies rather than rigid geopolitical boundaries.

Geographical Indications do not rely solely on strict soil or climate boundaries. They frequently recognize the reputation, traditional knowledge, and human factors tied to a broader region. The district of Besni is culturally and economically synonymous with extensive grape cultivation and specialized drying methods. The fact that the neighboring Gölbaşı district produces an identical physicochemical profile merely suggests that Gölbaşı shares the same agro‐ecological terroir and traditional agricultural practices as Besni, validating the wider regional cluster as a cohesive Geographical Indication zone. The Taguchi Signal‐to‐Noise (S/N) plots and Nested ANOVA cleanly isolate that while the macro‐region is flat and inactive, the drying method exerts a highly significant impact (*p* < 0.01) on the final product quality. Taguchi main effects plots, the slope for “Region” is nearly horizontal in both Graph A (incorporating color) and Graph B (excluding color). This visual flatness confirms the lack of a strong regional signal. Conversely, the “Drying” factor displays massive vertical slopes. Moving from Branch Drying (BB) to Sun Drying (BS/YS) radically alters the S/N ratio. This proves that a Besni Grape Raisin's ultimate quality identity is determined by human processing interventions rather than the strict geographical boundary line of the vineyard.

The Taguchi optimization method was employed to determine the most optimal grape variety and geographical region by taking into account all physical and chemical parameters. This method is an effective optimization approach that considers multiple parameters and is applicable in experimental designs with orthogonal arrays based on only two main factors (Ahmad et al. 2020). In this study, region and drying methods were considered as the two primary factors, and the most optimal factor levels were determined using eight measurement parameters based on the signal‐to‐noise (S/N) ratio, applying the ‘Nominal is Best’ criterion (10 × Log10(Y^2^/s^2^)).

**FIGURE 3:**
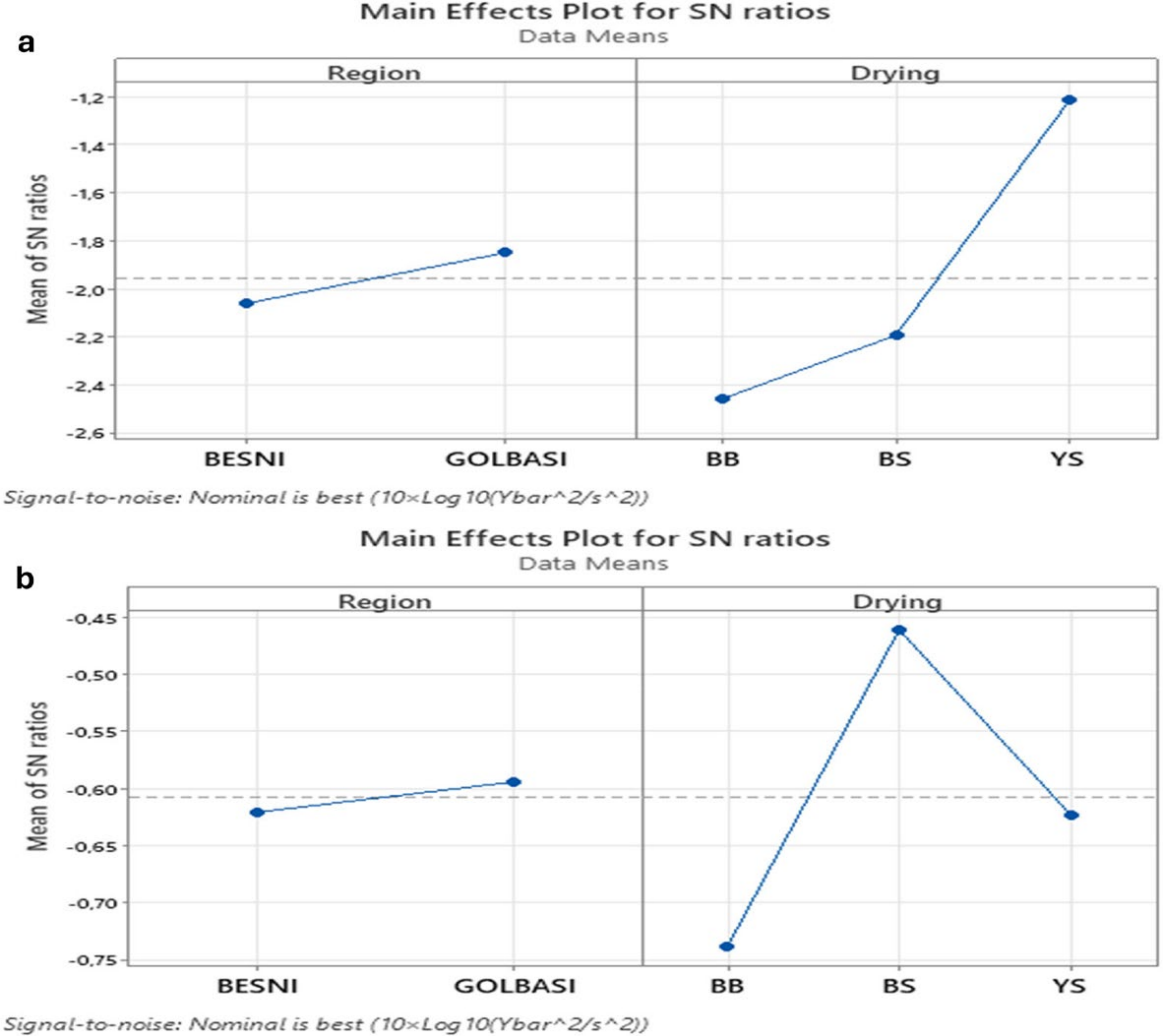
Signal to noise ratio effect graph for Taguchi optimisation method. (a) Graph containing pH, Brix, titratable acidity, and moisture (no color values). (b) Graph containing pH, Brix, titratable acidity, moisture, L*, a*, and b*. BBB, Besni Black Branch; BBS, Besni Black Sun; BYS, Besni Yellow Sun; GBB, Golbası Black Branch; GBS, Golbası Black Sun; GYS, Golbası Yellow Sun.

As demonstrated in Figure 3, in a manner analogous to the PCA graphs, two distinct S/N plots are presented. The graph on the ‘a’ displays the S/N results based on all physical and chemical analysis parameters applied to the grape samples. In contrast, the graph on the right excludes the Hunter L*, a*, and b* color values from the analysis. In the Taguchi optimization analysis excluding color values, the influence of geographical region appears minimal in both the left and ‘b’ S/N graphs in Figure 3. In the course of the present study, the three different drying methods were evaluated. The left S/N graph, which is based on the inclusion of Hunter L*, a*, and b* color values, shows that the most optimal drying method corresponds to the grape samples dried under yellow sunlight. Conversely, the right S/N graph (with the Hunter color values excluded) indicates that the most optimal drying method is associated with the grapes dried under black sunlight. These findings suggest that, in a manner analogous to the PCA analysis, the Hunter L*, a*, and b* color values exert a significant influence on the drying method.

In the Taguchi optimization analysis excluding color values, the influence of geographical region appears minimal in both the left and ‘b’ S/N graphs in Figure 3. In the course of the present study, the three different drying methods were evaluated. When interpreting these chemometric optimizations, a specific methodological constraint must be acknowledged: the absolute absence of yellow branch‐dried baseline matrices (BYB and GYB). Because branch‐drying was successfully completed only for the black variants, the orthogonal matrix treats the ‘Drying’ factor levels as asymmetrical across pigmentation profiles. While the Taguchi Signal‐to‐Noise (S/N) criterion remains robust under these specific group behaviors, this restriction limits the model's capacity to predict interactive metabolic shifts unique to yellow cultivars under shaded vine‐dehydration paths.

### 3.6 Analysis Results of Aroma Compounds

A total of 73 volatile aroma compounds were identified in the dried grape samples using the headspace solid‐phase microextraction (HS‐SPME) method combined with gas chromatography–mass spectrometry (GC–MS) for the extraction of free aroma substances. The most abundant aroma compounds detected in the grape samples are presented in Table 6. Among these, 5‐(Hydroxymethyl)furan‐2‐carbaldehyde (HMF), ethyl ethanoate, and propane‐1,2,3‐triyl triacetate were found in all grape varieties. The HMF content ranged between 18.7 and 35.23 mg/kg. The major concern for HMF is related to its bioactive derivative 5‐sulfoxymethyl‐2‐furfural that has been stated as a mutagenic compound (Gül et al. 2016). According to the Turkish Food Codex, the HMF limit values for liquid and solid grape molasses are 75 and 100 mg/kg, respectively Turkish Food Codex (2007). An Acceptable Daily Intake (ADI) value for Furan‐2‐carbaldehyde was established as 0.5 mg/kg body weight by the European Food Safety Authority (EFSA), but no references are written for HMF or others (Gül et al. 2016). Wang et al. (2023) stated that pre‐harvest benzothiadiazole treatment significantly reduced the free alcohol content of Cabernet Gernischt grapes and increased the levels of both free and bound esters.

**TABLE 6:** Flavor components of yellow and black Besni and Gölbaşı grape samples dried with different treatments.

Components (%)	Besni grape			Golbasi grape		
	BBB	BBS	BYS	GBB	GBS	GYS
5‐ (Hydroxymethyl) furan‐2‐carbaldehyde (HMF)	34.85	21.99	29.97	35.23	23.87	18.7
Ethyl ethanoate	7.73	6.43	4.14	6.17	6.19	5.66
Propane‐1,2,3‐triyl triacetate	4.81	5.01	3.6	4.47	4.26	7.12
Ethyl 2‐oxopropanoate	0.9	1.18	—	0.33	0.84	1.3
Pent‐1‐en‐3‐one	—	1.31	0.74	1.25	0.95	1.78
(2‐Methyl‐4‐oxopyran‐3‐yl) 2‐methylpropanoate	—	4.69	5.23	4.84	3.89	5.56
Hexadecanoate acid	—	0.48	1.56	0.93	0.91	3.86
Furan‐2‐carbaldehyde	5.99	—	8.46	7.31	2.85	—
Methyl 2‐methylpropanoate	1.84	2.21	—	—	1.98	3.01
5‐Methylfuran‐2‐carbaldehyde	1.77	—	0.08	0.59	0.28	—
Butyl acetate	1.14	—	0.7	0.83	0.98	1.17
(Tetrahydrofuran‐2‐yl)methyl butanoate	2.52	3.12	4.6	3.93	3.37	—
3,4‐Dimethylcyclopentane‐1,2‐dione	2.46	1.47	1.5	1.52	2.61	—
4‐Methylnonanoic acid	10.06	9.84	12.48	11.03	9.06	—
4‐Methylcyclohexan‐1‐one	—	0.69	0.41	0.53	—	0.8
3,7‐Dimethyloctan‐3‐ol	0.07	0.96	—	0.29	—	1.17
5‐Hydroxyoctan‐4‐one	—	0.54	—	—	0.53	0.63
Hexyl ethanoate	—	3.07	6.6	—	7.98	3.35
4‐Methylpentanoic acid	4.65	—	8.02	6.55	8.8	—
7‐Hexadecen‐16‐olide	0.92	—	2.25	0.06	1.81	—
3‐Mercaptobutan‐2‐one	—	13.05	5.64	—	—	13.97
4‐Hydroxy‐2,5‐dimethylfuran‐3‐one	—	0.39	—	0.43	0.3	—
Nonyl ethanoate	—	8.72	—	1.95	—	—
4‐Methoxy‐2,5‐dimethylfuran‐3‐one	1.24	—	—	—	—	1.06
Propyl formate	3.04	—	—	1.66	—	0.59
Methyl 3‐hydroxyhexanoate	—	2.17	1.44	1.32	—	—
5‐Methylthiophene‐2‐carbaldehyde	1.79	2.54	—	—	—	2.66
Octyl octanoate	0.09	—	—	0.17	0.33	—
Propyl formate	—	—	1.22	1.84	—	—
Furan‐2‐ylmethanol	—	1.29	—	—	—	—
2‐Methyltetrahydrofuran‐3‐one	—	0.17	—	—	0.27	—
Hexanedioic acid	3.07	—	—	—	0.73	—
Cyclohexanone	—	—	—	—	—	0.5
4‐Methylcyclohexan‐1‐one	0.56	—	—	—	0.63	—
Hexyl 3‐methylbutanoate	—	—	—	—	1.86	1.01
Isobutyl 3‐oxobutanoate	1.02	1.1	—	—	—	—
(E)‐6,10‐Dimethylundeca‐5,9‐dien‐2‐one	—	1.35	—	—	1.67	—
4‐Methyloctanoic acid	—	3.69	—	—	—	4.68
(1aR,4S,4aS,7R,7aS,7bS)‐1,1,4,7‐tetramethyldecahydro‐1H‐cyclopropa[e]azulene‐4a‐ol	0.11	—	0.6	—	—	—
1‐Methyl‐4‐(propan‐2‐yl)‐7‐oxabicycloheptane	0.15	—	—	0.1	—	—
Ethyl hexadecanoate	—	—	—	—	—	2.55
Ethyl octadecanoate	—	—	—	—	—	0.54
Oxacyclohexadecan‐2‐one	—	—	—	—	—	7.17
Ethyl 2‐hydroxypropanoate	—	—	—	0.35	—	—
3‐Methylbutanoic acid	—	—	—	—	5.57	—
Corillon	—	0.05	—	—	—	—
4‐Ethyloctanoic acid	—	—	—	—	0.88	—
(2,5‐Dimethyl‐3‐oxo‐2,3‐dihydrofuran‐4‐yl) acetate	—	2.49	—	—	—	—
Pentyl furan‐2‐carboxylate	2.6	—	—	—	—	—
Pentanal	—	—	—	—	—	0.83
(E)‐2‐Methylbut‐2‐enal	—	—	—	2.96	—	—
1‐(5‐Methylfuran‐2‐yl)ethan‐1‐one	—	—	—	1.59	—	—
5‐Propyloxolan‐2‐one	—	—	—	—	4.66	—
3‐Methylbut‐2‐en‐1‐yl acetate	—	—	—	—	1.94	—
4‐(Methylsulfanyl)butan‐2‐one	—	—	—	—	—	4.27
Oct‐1‐en‐3‐yl acetate	—	—	—	—	—	1.13
4‐Methylbenzaldehyde	2.88	—	—	—	—	—
Allyl 2‐(pentyloxy)acetate	—	—	—	—	—	0.54
(Tetrahydrofuran‐2‐yl) methyl acetate	—	—	—	—	—	1.55
Dodecyl acetate	—	—	—	—	—	1.88
2‐(4‐Methyl‐1,3‐thiazol‐5‐yl)ethyl acetate	—	—	0.76	—	—	—
Propyl heptanoate	—	—	—	1.11	—	—
Hexyl octanoate	2.66	—	—	—	—	—
3,5‐Dimethylcyclopentane‐1,2‐dione	0.23	—	—	—	—	—
(1R,4S)‐8‐Mercapto‐p‐menthan‐3‐one	—	—	—	0.15	—	—
Ethyl 3‐cyclohexylpropanoate	0.15	—	—	—	—	—
Furan‐2‐ylmethyl heptanoate	0.7	—	—	—	—	—
3,7‐Dimethyloct‐6‐en‐1‐yl acetate	—	—	—	0.09	—	—
Butyl acetate	—	—	—		—	0.45
2,6,6‐Trimethylcyclohex‐2‐ene‐1,4‐dione	—	—	—	0.17	—	—
2,6,10,10‐Tetramethyl‐1‐oxaspiro[4.5]dec‐6‐en‐8‐ol	—	—	—	0.14	—	—
(1S,4R,5R)‐4‐Methyl‐1‐(propan‐2‐yl)bicyclichexan‐3‐one	—	—	—	0.11	—	—
(1R,2S,5R)‐5‐Methyl‐2‐(prop‐1‐en‐2‐yl)cyclohexan‐1‐ol	—	—	—			0.51

Abbreviations: BBB, Besni Black Branch; BBS, Besni Black Sun; BYS, Besni Yellow Sun; GBB, Golbası Black Branch; GBS, Golbası Black Sun; GYS, Golbası Yellow Sun.

A total of 73 volatile aroma compounds were identified and monitored across the different raisin treatments. While the manuscript notes that individual major markers like hydroxymethylfurfural (HMF), ethyl acetate, and triacetin were omnipresent, a deeper classification of these volatiles reveals distinct shifts in chemical groups—predominantly esters, aldehydes/furans, volatile organic acids, ketones, and terpenoids—driven by processing conditions.

Ester: Esters represent one of the most structurally diverse classes detected in the samples, heavily influencing the desirable sweet and fruity background aroma of the raisins. Dominant Compounds: Ethyl ethanoate (ethyl acetate), propane‐1,2,3‐triyl triacetate (triacetin), hexyl ethanoate, and butyl acetate. Ethyl ethanoate was consistently preserved across all samples, peaking slightly in Besni Black Branch (BBB) at 7.73%. Interestingly, complex matrix transformations or concentration effects significantly boosted triacetin in Gölbaşı Yellow Sun (GYS) to 7.12%. Longer‐chain or branched esters like hexyl ethanoate were entirely undetected in branch‐dried samples (BBB, GBB) but emerged strongly under sun drying, particularly in Gölbaşı Black Sun (GBS) at 7.98%. This indicates that direct sun exposure and the associated thermal kinetics favor the esterification of mid‐chain alcohols and organic acids during the dehydration process.

Volatile Fatty Acids: Volatile organic acids accumulate via lipolytic degradation or carbohydrate metabolism disintegration during post‐harvest stress. Dominant Compounds: 4‐Methylnonanoic acid, 4‐Methylpentanoic acid, and 3‐Methylbutanoic acid. 4‐Methylnonanoic acid was prominently high in almost all black grape treatments, holding steady around 10.06% to 12.48% in BBB, BBS, BYS, and GBB. However, it was completely absent in the yellow sun‐dried Gölbaşı grapes (GYS). The appearance of 3‐Methylbutanoic acid uniquely in GBS (5.57%) highlights that open sun drying of black grapes sets up an oxidative environment that triggers specific amino acid degradation or lipid oxidation pathways not favored on the branch.

Ketones and Sulfur‐Containing Volatiles: Ketones contribute heavily to fatty, toasted, or pungent aromatic sub‐notes. Dominant Compounds: 3‐Mercaptobutan‐2‐one, 3,4‐Dimethylcyclopentane‐1,2‐dione, and (2‐Methyl‐4‐oxopyran‐3‐yl) 2‐methylpropanoate. A standout observation is the massive presence of the sulfur‐containing ketone 3‐Mercaptobutan‐2‐one in sun‐dried samples. Besni Black Sun (BBS) at 13.05% and Gölbaşı Yellow Sun (GYS) at 13.97%. Remarkably, this compound was completely undetected in branch‐dried grapes (BBB, GBB). Ultraviolet radiation from direct sun exposure likely drives photochemical reactions involving sulfur‐containing amino acids (like methionine or cysteine), generating distinct volatile sulfur compounds that drastically alter the sensory profile from vine‐dried counterparts.

Terpenoids and Norisoprenoids: Terpenes provide floral and balsamic notes and represent the fundamental varietal aroma baseline of the *Vitis vinifera* species. Dominant Compounds: 3,7‐Dimethyloctan‐3‐ol, (E)‐6,10‐Dimethylundeca‐5,9‐dien‐2‐one, and various cyclic oxides. Terpenoids generally appeared in very low relative percentages or were completely bound up/degraded by intense sun drying. For instance, 3,7‐Dimethyloctan‐3‐ol was preserved in minor amounts in BBB, BBS, GBB, and GYS. The harsh thermal conditions of direct sun exposure usually cause the evaporation or oxidative degradation of delicate free monoterpenes, leaving behind heavier, heat‐stable secondary derivatives.

This chemical distribution aligns perfectly with our PCA findings. The clustering of BBB and GBB on the PCA plot is largely steered by their high concentration of thermal furan derivatives (HMF and furfural), while the sun‐dried variants (BBS, GBS, GYS) scatter towards regions dominated by newly generated volatile esters, fatty acids, and sulfurous ketones (e.g., 3‐mercaptobutan‐2‐one) generated via active solar radiation.

Karadeniz et al. (2000) reported HMF levels in sun‐dried raisins as 53.4 mg/kg, in dipped raisins as 85.5 mg/kg, and in frozen grapes as 0.3 mg/kg, noting that no HMF was detected in fresh grapes (Karadeniz et al. 2000). In a similar vein, Gül et al. (2016) measured HMF levels at 3.59 mg/kg in packaged raisins and 12.84 mg/kg in unpackaged raisins. It is important to note that HMF is not a naturally occurring compound in fruits and vegetables. Instead, it is formed during the Maillard reaction, which is a non‐enzymatic browning process that occurs during the drying of food products. This reaction has been shown to have a detrimental effect on the quality and sensory characteristics of the product (Cesur 2013).

**FIGURE 4:**
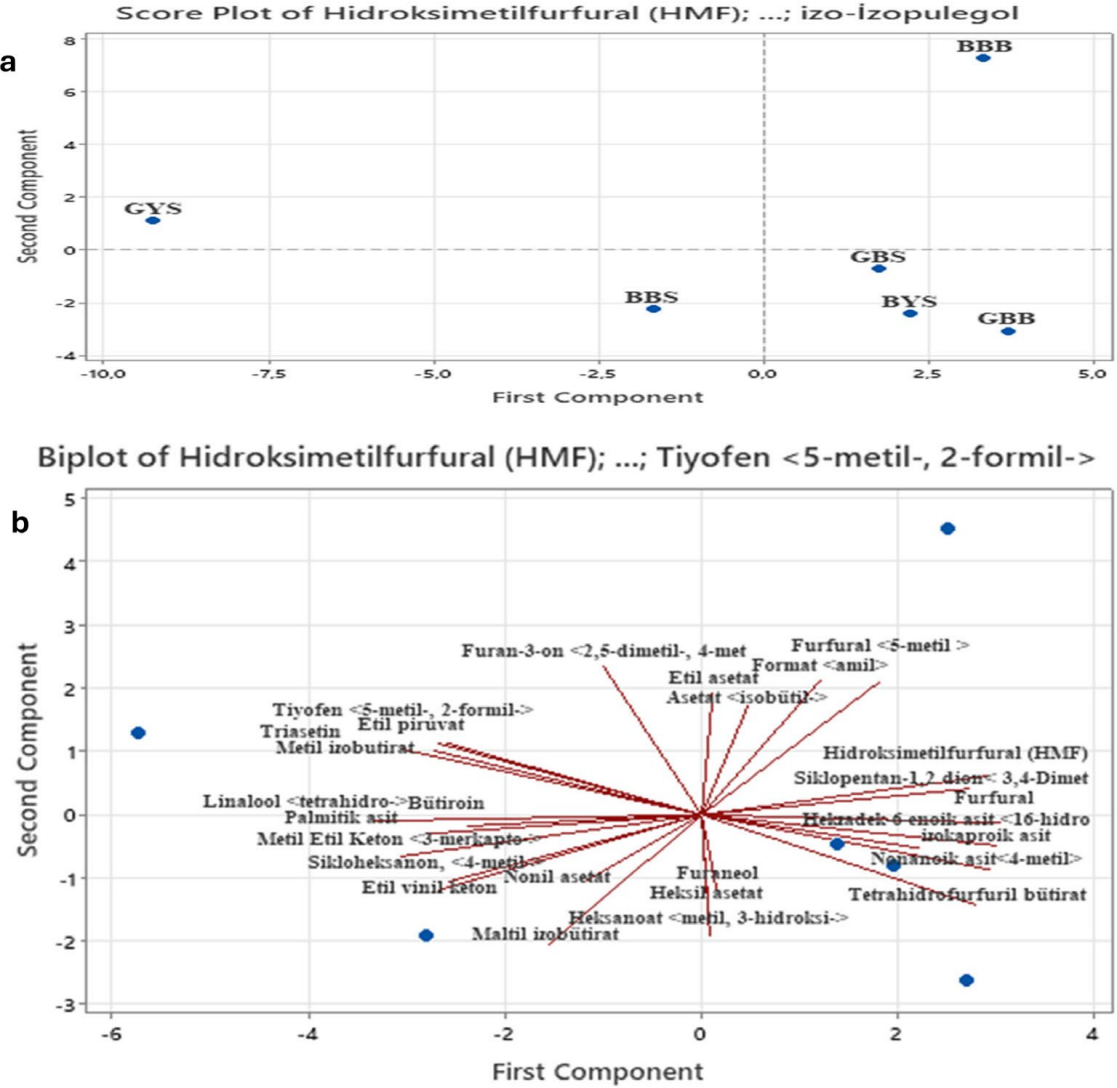
Aroma components principal component analysis. BBB, Besni Black Branch; BBS, Besni Black Sun; BYS, Besni Yellow Sun; GBB, Golbası Black Branch; GBS, Golbası Black Sun; GYS, Golbası Yellow Sun. (a) Score plot graph of PCA analysis; (b) Biplot graph of PCA analysis.

As demonstrated in the PCA graphs in Figure 4, the BBB‐dried, BBS‐dried, and GYS‐dried grape samples are distinctly positioned away from the other grape samples. Furthermore, these two samples are significantly distant from each other. Other grape samples generally showed close clustering. Seventy‐three parameters were reduced to four components by a PCA, and four components explained 89.4% of total variance. The graph's biplot analysis displays the 27 most commonly detected aroma compounds in the grape samples. Generally, aroma compounds are found in the region where the BBB‐dried, BBS‐dried, and GYS‐dried grape samples are located, while the most abundant aroma compound, HMF, is found in the region where the other grape samples are located.

### 3.7 ATR‐FTIR Analysis Results

In the domain of food analysis, Attenuated Total Reflectance Fourier Transform Infrared (ATR‐FTIR) spectroscopy has been identified as a potential instrument for both qualitative and quantitative quality control assessment. FTIR‐ATR provides a straightforward and reproducible method for analyzing both liquid and solid samples, with analyses conducted without subjecting the samples to chemical treatment, following a brief (less than 5 min) physical sample preparation process. The combination of ATR‐FTIR techniques with chemometric methods has been demonstrated to be a successful approach for the detection of adulteration and imitation in various products, including fruit purées, honey, and oils (Irudayaraj et al. 2003; Ozen et al. 2003; Vardin et al. 2008).

**FIGURE 5:**
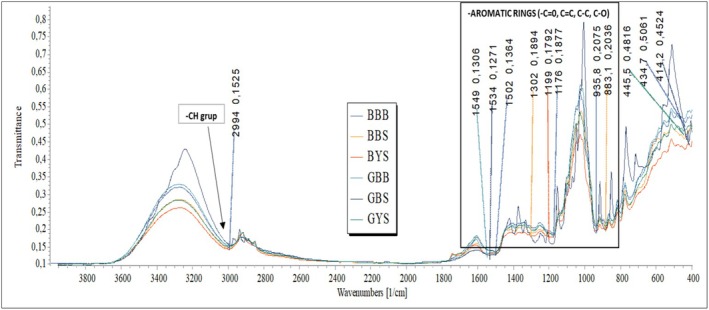
ATR‐FTIR spectra of yellow and black Besni and Gölbaşı grape samples dried with different treatments.

In this study, the ATR‐FTIR spectra of yellow and black Besni and Gölbaşı grape samples dried using different methods are presented in Figure 5. A thorough examination of the obtained spectra revealed that the sun‐dried black Gölbaşı grape variety exhibited significantly different spectral peaks compared to the other grape varieties. These disparities were discernible across a broad spectrum of wavelengths. In contrast, although some variations were observed in the spectral peaks of the other grape varieties, their spectra were generally like each other. BBB and GBB dried grapes differed from BBS, BYS, and GYS dried grapes in the spectral region range of 1000 to 1100 cm^−1^ and 2900 to 2950 cm^−1^. These differences are believed to arise from functional groups such as C—H and C=C aromatic rings, O—H bonds, C=O (aldehyde, ketone), and C–O and C—C bonds. Yaman (2019) associated the broad band observed around 3292 cm^−1^ and the band at 1626 cm^−1^ in grape samples with O—H stretching and O—H deformation, respectively, while the peaks observed around 2908 cm^−1^ were related to the C—H stretching of carboxylic acids.

### 3.8 Partial Least Squares‐Regression Analysis (PLS‐R)

In the realm of chemometric calibration methodologies, Partial Least Squares Regression (PLS‐R) stands out as a particularly prevalent and frequently employed technique. PLS‐R is a linear model based on two data sets: the spectral data and the target parameter to be determined (Zhang et al. 2017). The evaluation criteria for the calibration model encompass the correlation coefficient of calibration (Rc), the root mean square error of calibration (RMSEC), the correlation coefficient of cross‐validation (Rcv), and the root mean square error of cross‐validation (RMSECV). RMSEC is indicative of the extent to which a model aligns with the calibration dataset, while RMSECV signifies the predictive capacity of the model within the calibration set and aids in determining the optimal number of latent variables. In a model that exhibits optimal performance, the Root Mean Square Error of Cross‐Validation (RMSECV) and the Root Mean Square Error of Calibration (RMSEC) should be minimal, while the Rc and Rcv parameters should be substantial. The discrepancy between RMSEC and RMSECV is expected to be negligible.

The evaluation of the predictive performance of the calibration model is facilitated by the Residual Predictive Deviation (RPD). RPD is defined as the ratio of the standard deviation (SD) to the root mean square error of the central tendency (RMSECV). A higher RPD value is indicative of a greater capability of the calibration model to predict chemical composition in the samples. Typically, an RPD value exceeding 5 is deemed adequate for quality control purposes, whereas values surpassing 3 are regarded as exceptional for prediction applications. However, in most applications, an RPD below 1.5 is deemed inadequate, whereas values above 2.0 are considered acceptable and useful for screening purposes (Qin et al. 2015).

In the mid‐infrared fingerprint region (800–1800 cm^−1^), the optimal PLS‐R calibration architectures required the extraction of six to nine latent variables (factors), capturing 92.08%–96.31% of the total spectral variance. Retaining nine factors for highly complex parameters like titratable acidity is mathematically justified by the cross‐validation metrics (*R* ^2^ = 0.825, RMSECV = 0.144), which demonstrate that the model successfully captures real, multi‐component chemical variations without succumbing to rank‐overinflation. The fingerprint zone contains highly dense, overlapping vibrational bands stemming from simultaneous C=O stretching, C–O stretching, and skeletal carbohydrate ring deformations that require a higher factor dimensionality to isolate the specific acidity signature from the shifting sugar matrix.

However, a strict framing threshold was applied to evaluate model generalizability: any calibration model yielding a cross‐validation coefficient (*R* ^2^) below 0.50 is classified as fundamentally unstable and completely unsuitable for quantitative prediction or screening applications. Consequently, while the high‐wavenumber region (2800–3800 cm^−1^) delivered an exceptional, parsimonious model for titratable acidity using only 5 factors (*R* ^2^ = 0.915), physical parameters like color (*R* ^2^ = 0.327 for L*) and highly sensitive equilibrium metrics like pH (*R* ^2^ = 0.354) collapsed entirely below this 0.50 threshold. This mathematical failure confirms that high factor retention in these specific poor‐performing models merely captured localized baseline noise or scattering artifacts rather than authentic chemical phenomena, rendering them invalid for industrial deployment.

In the present study, all samples were used to construct the calibration model, and the cross‐validation method was employed to evaluate the prediction accuracy and reliability of the PLS‐R method. For the Besni and Gölbaşı grape samples, which are characterized by yellow and black pigmentation respectively, the application of different drying methods yielded divergent results. The employment of second derivative transformations of normalized spectral data in the infrared regions of 800–1800 cm^−1^ and 2800–3800 cm^−1^ was found to yield superior outcomes. Consequently, in accordance with the study conducted by Nizamlıoğlu et al. (2022), the second derivative of the normalized data was employed. Spectral derivatives are designed to remove baseline shifts, resolve overlapping peaks, and accentuate subtle chemical features.

A total of 30 ATR‐FTIR spectra from the dried grape samples were analyzed for the PLS‐R models: 24 spectra were assigned to the calibration set, while the remaining 6 spectra were reserved as an independent validation set to rigorously assess the predictive power of the methodology. Rather than utilizing simple random selection at the individual spectral scan level, this partitioning was executed strictly at the biological sample level as indivisible blocks. This group‐blocked allocation guaranteed that all technical replicates from a given biological sample remained together, validating the model against entirely unseen biological profiles. The 6 validation spectra were carefully partitioned to be representative of the entire dataset while ensuring that extreme value boundaries (minimum and maximum outliers) remained within the calibration matrix to preserve model stability. Mid‐IR Fingerprint Region (800–1800 cm^−1^): This region captures the fundamental vibrations of major organic components (carbohydrates, proteins, and organic acids). The models use 6–9 factors, capturing a very high percentage of the spectral variance (92.08%–96.31%). This indicates that the selected latent variables are highly representative of the chemical matrix changes. The calibration models are robust, with excellent fits (*r* ^2^ ranging from 0.893 to 0.985) and low RMSEC values. However, when looking at cross‐validation (*R* ^2^ and RMSECV), we see a noticeable drop in performance across all parameters, with *R* ^2^ hovering between 0.759 and 0.825. Color Parameters (L*, a*, b*): While calibration statistics look phenomenal (*r* ^2^ > 0.97), the RMSECV values jump significantly (e.g., 53.78 for L*). This indicates that while the model learns the calibration set well, its prediction error on unseen data is high, suggesting potential overfitting or high sensitivity to baseline shifts in this region. Acidity and pH: Titratable Acidity (TA) shows the most balanced and reliable performance in this region (*r* ^2^ = 0.967, *R* ^2^ = 0.825, and a relatively low validation error of 0.144) (Table 7). This is consistent with the fact that organic acid C=O and C—O stretching bands are highly active in this fingerprint zone.

**TABLE 7:** Calibration and validation results of PLS‐R models of quality characteristics of yellow and black Besni and Gölbaşı grape samples dried with different treatments in the infrared spectral range of 800–1800 cm^−1^ and 2800–3800 cm^−1^.

Components	Factor	Variance (%)	*r* ^2^	RMSEC	*R* ^2^	RMSECV	*p*
800–1800 cm^−1^							
pH	9	96.31	0.968	0.015	0.759	0.113	0.000
Titratable Acidity	9	96.31	0.967	0.028	0.825	0.144	0.000
Brix	6	92.08	0.901	18.710	0.759	45.441	0.000
Moisture	6	92.08	0.893	1.708	0.776	3.594	0.000
L*	9	96.31	0.979	5.059	0.780	53.783	0.000
a*	9	96.31	0.985	2.014	0.811	25.415	0.000
b*	9	96.31	0.974	8.259	0.800	63.288	0.000
2800–3800 cm^−1^							
pH	5	72.61	0.819	0.085	0.354	0.303	0.000
Titratable Acidity	5	72.61	0.953	0.039	0.915	0.070	0.000
Brix	5	72.61	0.919	15.277	0.748	47.614	0.000
Moisture	5	72.61	0.905	1.530	0.827	2.768	0.000
L*	5	72.61	0.851	36.496	0.327	164.757	0.000
a*	5	72.61	0.850	20.088	0.542	61.512	0.000
b*	5	72.61	0.870	47.158	0.447	174.612	0.000

*Note:* Factor: Number of latent variables; $r^{2}$: Coefficient of determination for calibration; RMSEC: Root mean square error of calibration; $R^{2}$: Coefficient of determination for cross‐validation; RMSECV: Root mean square error of cross‐validation. Data partitioning for the calibration set (n=24) and independent validation set (n=6) was performed strictly using a group‐blocked strategy at the biological sample level. All technical replicates of any single biological sample were held together as an indivisible unit within the same set, ensuring that technical duplicates did not cross between calibration and validation matrices, thereby preventing data leakage.

High‐Wavenumber Region (2800–3800 cm^−1^): This region is dominated by O—H stretching (water/carbohydrates) and N—H/C—H stretching bands. Interestingly, TA performs exceptionally well here. It achieves an *R* ^2^ of 0.915 and a remarkably low RMSECV of 0.070 (which is lower than its RMSEC of 0.144 in the fingerprint region). The lower factor count (5 vs. 9) combined with a high cross‐validation *R* ^2^ indicates a highly robust, parsimonious, and generalizable model for acidity in this region. Moisture and Brix: Both show steady, reliable predictability. Moisture benefits from the strong O—H stretching water bands in this region, yielding a solid *R* ^2^ of 0.827 and a low RMSECV of 2.768. Poor Performers (pH and Color): pH collapses completely in this region (*R* ^2^ = 0.354, RMSECV = 0.303), meaning the model cannot reliably predict pH beyond random variation. Color parameters (L, a*, and b*) exhibit very low *R* ^2^ values (down to 0.327 for L*) and massive prediction errors (RMSECV up to 174.6) (Table 7). This region is largely unsuitable for monitoring physical color attributes. The 2800–3800 cm^−1^ region yields a far superior and more robust model than the fingerprint region, despite explaining less overall spectral variance. It uses fewer factors and has a much higher prediction success rate (*R* ^2^ = 0.915). Both regions offer acceptable predictive power (*R* ^2^≈0.75–0.82). However, moisture performs slightly better in the higher wavenumber region (*R* ^2^ = 0.827) due to direct O—H water band interactions. The 800–1800 cm^−1^ region is mandatory if you intend to model these parameters. They fail completely at higher wavenumbers. However, be cautious of the high RMSECV values in the fingerprint region, which suggest you may need to apply spectral preprocessing (like Standard Normal Variate (SNV) or Multiplicative Scatter Correction (MSC)) to minimize those prediction errors. All models across both regions are highly statistically significant (*p* = 0.000). While the integration of ATR‐FTIR spectroscopy and PLS‐R chemometrics demonstrates strong performance for the rapid, chemical‐free prediction of moisture content and titratable acidity in these specialized raisin matrices, certain constraints must be acknowledged before industrial deployment can be realized. The primary limitation of the current calibration models stems from the narrow and highly controlled sample population utilized, which was strictly constrained to specific traditional varieties harvested within a single production season across two adjacent districts in the Adıyaman province. Dehydrated food matrices like raisins exhibit considerable chemical and structural plasticity; fluctuations in annual meteorological conditions, ambient solar irradiance, soil chemistry, and localized vineyard management practices can introduce significant variations in the baseline sugar‐to‐acid ratios, cuticular wax thicknesses, and secondary pigment profiles of the raw material. A calibration model built on a localized sample cohort may suffer from limited robustness or overfitting when exposed to external, unmodeled matrix variations. To transform this methodology into a rugged, universally applicable commercial tool for automated quality monitoring on processing lines, further expansion of the calibration database is strictly required. Future research will focus on scaling the model by continuously incorporating independent biological samples collected across multiple harvest years, diverse macro‐climatic regions, and varying commercial agricultural practices. Broadening the population density in this manner will encapsulate a wider variance of infrared spectral profiles, thereby enhancing the predictive accuracy, reducing validation errors, and ensuring the long‐term robustness necessary for real‐time industrial sorting and quality control applications.

## 4 Conclusion

The findings of this study indicated that the pH values of Gölbaşı grapes were significantly higher (*p* < 0.05), and that direct sun‐drying exerted a distinctive effect on pH, particularly in black grape varieties. The titratable acidity levels of the grapes from Gölbaşı and Besni were found to be very similar, indicating that geographic origin did not significantly affect titratable acidity. The results demonstrated that the Gölbaşı exhibited higher °Brix values and moisture content than the Besni. Furthermore, a significant disparity in the color values of the grapes from the Gölbaşı region was observed when compared to those from the Besni region. Specifically, the Gölbaşı grapes demonstrated higher values for L*, a*, and b*, indicating a more pronounced color intensity and variation. Among the yellow grape varieties, those dried in direct sunlight exhibited significantly higher L*, a*, and b* values (*p* < 0.05). In black grape varieties, no statistically significant differences were found in color values between sun‐dried and on‐vine dried samples.

The findings of the Principal Component Analysis (PCA) and the Taguchi optimisation method, which were conducted on the physical components of the grape samples, indicated that the Hunter L*, a*, and b* color values exhibited a substantial influence on the physical and chemical properties of the dried grapes. The results of the nested analysis of variance (Nested Model) applied to evaluate the effects of region and drying methods indicate that the impact of geographic origin on all grape components is negligible. However, it is evident that divergent drying methods exert a substantial influence on product quality. The HS‐SPME method was employed for the extraction of free aroma compounds, and the GC–MS technique was used for analysis. This approach enabled the identification of a total of 73 volatile aroma compounds. The presence of 5‐(Hydroxymethyl)furan‐2‐carbaldehyde (HMF), ethyl ethanoate, and propane‐1,2,3‐triyl triacetate was detected in all varieties of grape. The results of the Nested Model Analysis of Variance revealed that while divergent post‐harvest drying methods exert a highly significant influence on final product quality (*p* < 0.01), the direct impact of geographical origin across the neighboring Besni and Gölbaşı districts remains statistically negligible (*p* > 0.05). This outcome introduces a notable chemometric paradox, demonstrating that standard physical and chemical parameters cannot be utilized as distinctive standalone markers to differentiate the two tested regions. Nevertheless, this lack of regional variance mathematically establishes a highly uniform baseline terroir across these adjacent borders. It underscores that the definitive quality attributes and typicity of the geographically indicated Besni Grape Raisin are fundamentally driven by shared traditional post‐harvest human interventions—specifically on‐branch versus direct sun dehydration workflows—rather than strict geographical boundary constraints.

The spectrochemometric modeling demonstrated that the selection of the infrared wavenumber range plays a pivotal role in the prediction accuracy of the developed Partial Least Squares Regression (PLS‐R) models. Based on the low RMSEC and RMSECV values, minimal error disparities, and high R2 values, the PLS‐R model utilizing the 800–1800 cm^−1^ spectral range proved to be a highly suitable and accurate tool for predicting the moisture content of the raisins. Conversely, for titratable acidity analysis, the PLS‐R model utilizing the higher 2800–3800 cm^−1^ spectral range delivered superior performance, yielding highly precise and adequate quantitative predictions. These findings confirm that non‐destructive ATR‐FTIR spectroscopy integrated with targeted PLS‐R chemometrics provides a robust, chemical‐free path for monitoring critical quality parameters in geographically indicated Besni grape raisins.

## Author Contributions

**Fatma Erişik:** conceptualization, investigation, resources, methodology. **Nizam Mustafa Nizamlioğlu:** writing – review and editing. **Yalçın Güçer:** formal analysis, data curation, supervision, methodology, validation.

## Funding

The authors have nothing to report.

## Ethics Statement

The authors have nothing to report.

## Consent

All authors agreed to the publication of the manuscript.

## Conflicts of Interest

The authors declare no conflicts of interest.

## Data Availability

Data available on request from the authors.
